# Healthy Diets Are Associated with Weight Control in Middle-Aged Japanese

**DOI:** 10.3390/nu17193174

**Published:** 2025-10-08

**Authors:** Etsuko Kibayashi, Makiko Nakade

**Affiliations:** 1 Department of Food and Nutrition Management, Sonoda University, Amagasaki 661-8520, Hyogo, Japan; 2 Department of Food Science and Nutrition, University of Hyogo, Himeji 670-0092, Hyogo, Japan; 3 Research Institute for Food and Nutritional Sciences, Himeji 670-0092, Hyogo, Japan

**Keywords:** middle age, healthy diets, weight control, eating behaviour, cross-sectional study

## Abstract

**Background/Objectives:** In Japan, well-balanced meals composed of staple grains, protein-rich main dishes, and vegetable sides are recommended. However, issues such as infrequent breakfast consumption and poor vegetable intake persist. Obesity and non-communicable disease (NCD) rates from age 40 have also begun rising. Therefore, we investigated the structural associations between healthy diets and weight control for NCD prevention, including the potential associations with rice consumption and eating out/home meal replacement use in middle-aged Japanese individuals. **Methods:** This study was a cross-sectional survey based on data from 577 respondents to the 2016 Hyogo Diet Survey, Hyogo Prefecture, Japan, aged 40–59 years. A healthy diet was defined as including at least two well-balanced meals daily, eating breakfast regularly, and eating five or more vegetable dishes daily. A hypothetical model included factors associated with healthy diets and maintaining a healthy weight (energy, salt, fat, and sugar intake; using nutritional fact labels; and regular exercise), and the frequencies of rice consumption and eating out/home-meal replacement. A simultaneous multi-population analysis by sex was performed. **Results:** Simultaneous multi-population analysis showed acceptable goodness-of-fit. Maintaining appropriate weight and eating rice were positively associated with healthy diet scores in both sexes. However, for men, using home meal replacements was negatively associated. **Conclusions:** Among middle-aged Japanese in Hyogo Prefecture, weight control for NCD prevention and rice consumption were linked to healthy diets. In men, using home meal replacements was associated with worse diet quality.

## 1. Introduction

Higher-than-optimal body mass index (BMI) was responsible for an estimated 5 million deaths from non-communicable disease (NCDs) in 2019, such as cardiovascular diseases, diabetes, cancers, and chronic respiratory diseases [1]. The obesity epidemic also has serious economic impacts. If nothing is done, the global costs of being overweight or obese are predicted to reach US$ 3 trillion per year by 2030 and more than US$ 18 trillion by 2060 [2]. The World Health Organization (WHO) reports that the risk of being overweight (BMI ≥ 25) or obese (BMI ≥30), as well as their related NCDs can, at the individual level, be reduced by adopting healthy eating behaviours. Such habits include limiting the consumption of sugar-sweetened beverages and energy-dense foods; limiting energy intake from total fats and sugars; increasing consumption of fruit and vegetables, as well as legumes, whole grains, and nuts; and engaging in regular physical activity [3]. In addition, dietary behaviours associated with preventing hypertension, such as restricting salt intake and consuming potassium-rich fruits and vegetables, are expected to contribute to preventing obesity [4]. Vegetable intake is reportedly higher among those who are aware of the benefits of preventing obesity [5]. The WHO also reports that stopping the rise in obesity demands policies and actions to create healthy food environments that make healthier food options available, accessible, and desirable [3]. Promoting nutritional labelling of foods and its use by consumers is one such example. Given these facts, practising common dietary behaviours and performing physical activity to maintain an appropriate weight, plus the use of nutritional fact labels, are key for preventing NCDs in middle-aged adults, who show rising incidences of obesity and NCDs.

In Japan, the recommended healthy diet includes well-balanced meals composed of staples (cereal grains), main dishes (proteins), and sides (vegetables). The risk of death, stratified by sex and age, from cardiovascular disease [6,7] and cerebrovascular disease [7] is lower in individuals who adhere to the daily recommended amounts of various food groups, as indicated in the 2005 Dietary Balance Guide [8]. The Japanese government has aimed to increase the proportion of Japanese individuals consuming well-balanced meals at least twice daily to at least 50% of the population by 2025, compared to the actual figure of 36.4% in 2020 [9].

In relation to NCDs, irregular breakfast intake [10] and missing breakfast [11,12] increase the risk of type 2 diabetes. In contrast, daily breakfast is beneficial in preventing stroke [13], and vegetable intake has preventive effects against chronic diseases such as cardiovascular disease [14], obesity [15] and cancer [16]. Therefore, healthy diets to prevent NCDs involve three deeply related dietary behaviours: a well-balanced diet, regular breakfast consumption, and the consumption of vegetables.

Many Asian countries traditionally eat rice as a staple food because rice cultivation is well suited to their climates and landscapes. In Japan, rice has been widely consumed since the Yayoi period (c. 300 BCE–300 CE). This has led to the development of ‘washoku’ (Japan’s traditional cuisine), which is known for its well-balanced nutrition centred on rice. The relationship between rice consumption and dietary balance has attracted attention outside of Asia, with 10 years of U.S. data showing that a high percentage of adults who consume rice tend to limit their intake of total fat and saturated fatty acids and choose diets rich in vegetables, dietary fibre, and iron, compared to those who do not consume rice [17]. Among Japanese people who eat rice three or more times a day, regardless of sex or age, many eat a complete meal including a staple, main dish, and side dishes at each meal [18], while eating rice for breakfast is associated with a well-balanced breakfast intake [19]. Thus, eating rice is expected to have a positive effect on healthy eating patterns.

Furthermore, when considering other eating behaviours related to healthy diets, the 2015 National Health and Nutrition Survey reported that those aged 20 years and older who regularly eat out and use home meal replacements (i.e., consumption of ready-to-eat foods) consumed well-balanced meals less frequently, and among those who consume daily meals combining staples, main and side dishes, vegetable-based side dishes were eaten the least [20]. Additionally, a previous study of Hispanics/Latinos reported that men eat lower-quality meals and more frequently eat meals that were not prepared at home than women [21]. Although many of these studies have indicated that weight management behaviours, rice consumption, and eating out or home meal replacement use are individually associated with healthy diets, no studies have simultaneously modelled these factors.

This study aimed to investigate the relationship between maintaining an appropriate weight to prevent NCDs and following a healthy diet, with a particular focus on the role of rice consumption. It also considered the impact of eating out and the use of home meal replacements on diet quality among middle-aged Japanese individuals, who show increasing prevalence of obesity and NCDs.

## 2. Materials and Methods

### 2.1. Study Design and Participants

This study examined secondary data from the 2016 Hyogo Diet Survey [22] and was approved by the Hyogo Prefectural Government. This survey was planned by the Health Promotion Division of the Hyogo Prefectural Government to ascertain the physical condition, nutrient intake, and lifestyle habits of prefectural residents and to provide basic data to comprehensively promote health. The study population consisted of 4747 individuals from 1919 households selected from 32 stratified random sampling districts within Hyogo Prefecture, based on the stratification criteria of the 2015 National Census [23]. This study used data from the 2016 National Health and Nutrition Examination Survey [24], which were provided in anonymised form for secondary analysis by the Hyogo Prefecture Health Promotion Division. As only secondary anonymised data were used, informed consent from participants was not required. Furthermore, since the anonymised data were not linkable, the study fell outside the scope of the Ethical Guidelines for Medical Research Involving Human Subjects [25]; thus, ethical review was also unnecessary.

### 2.2. Metrics

The survey collected participant characteristics including sex, age, living arrangement (living alone, married couple, parent[s] and children, 3- or 4-generation household, or other), and BMI. BMI was calculated from self-reported measurements as weight (kg)/height (m)^2^ and categorised as <18.5, 18.5–25, or ≥25 kg/m^2^.

Eating and lifestyle behaviours, and their interval scales, are shown in Table 1. The questionnaire items from the 2016 Hyogo Diet Survey were used for analysis. A healthy diet was defined as including at least two well-balanced meals daily, eating breakfast regularly, and eating five or more vegetable dishes daily [26]. A well-balanced meal was defined as one consisting of staples (cereal grains), main dishes (proteins), and sides (vegetables).

The factors considered important for maintaining appropriate weight to prevent NCDs were control of energy intake, restriction of salt intake, control of fat intake, control of sugar intake, use of nutritional fact labels, and regular exercise. Other items that potentially relate to healthy diets were the frequency of eating rice for breakfast, lunch, and dinner; frequency of eating out; and frequency of using home meal replacement (ready-to-eat food) (Table 1). For these items, scores were assigned in ascending order, with 1 point assigned to the lowest frequency and 4 to 7 points assigned to the highest frequency. The frequency of eating rice for breakfast, lunch, and dinner is the number of times (days) per week. For example, regardless of whether the amount of rice eaten for breakfast in one day is 100 g or 300 g, it is considered one time (one day).

### 2.3. Data Analysis

A total of 649 participants aged 40–59 years participated in the 2016 Hyogo Diet Survey. Among them, data from 577 participants (255 men and 322 women) without any missing items of data were included in this study. Sex-based comparisons of the ratios in terms of age group, living arrangement, and BMI were performed using the chi-squared test or Fisher’s exact test.

The scores for healthy diets (having a well-balanced meal at least twice daily, eating breakfast regularly, and number of vegetable dishes daily), maintaining appropriate weight (control of energy intake, restriction of salt intake, control of fat intake, control of sugar intake, use of nutritional fact labels, and regular exercise), frequency of eating rice, eating out frequency, and frequency of home meal replacement use were considered as interval scales, with sex and age group differences, as well as the absence of interaction effects among sex and age groups, being examined using a two-way analysis of variance.

**Figure 1:**
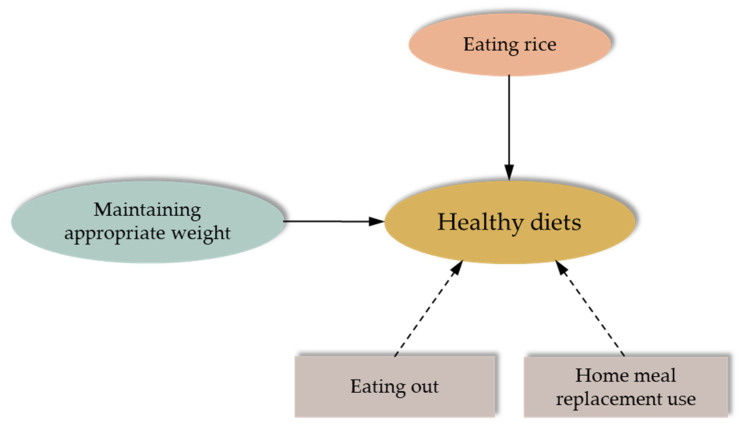
Initial hypothetical model of factors associated with healthy diets. The solid arrows indicate positive paths, while the dashed arrows indicate negative paths.

We developed an initial hypothetical model to investigate the structural associations of maintaining appropriate weight for NCDs prevention (controlling energy intake, restricting salt intake, controlling fat intake, controlling sugar intake, use of nutritional fact labels, and regular exercise) with healthy diets, including eating behaviours (eating rice, eating out, and home meal replacement use) potentially related to healthy diets (Figure 1). The goal of this analysis is to comprehensively understand the relationships among multiple variables. The initial hypothesised model was initially assessed by performing a structural analysis of covariance as part of men’s and women’s overall and structural examination. Subsequently, we conducted a simultaneous multi-population analysis according to sex to assess the model’s goodness-of-fit in each population and to examine construct invariance (to ascertain whether the model structure was consistent across groups) [27]. During this process, we repeatedly refined the model by removing statistically insignificant paths and other adjustments. The final hypothesis model was obtained by modifying the initial hypothesis model until the best fit was achieved. Sex differences in estimated values were evaluated through pairwise parameter comparisons. Model fit was assessed using indices such as the Goodness-of-Fit Index (GFI), Adjusted GFI (AGFI), Comparative Fit Index (CFI), Root Mean Square Error of Approximation (RMSEA), and Akaike’s Information Criterion (AIC). Path directions and standardised estimates were iteratively modified (e.g., by removing non-significant paths), and fit indices were refined until optimal fit was achieved. Model fit was judged adequate when GFI, AGFI, and CFI exceeded 0.9 and RMSEA was ≤0.05. The required sample size was determined using RMSEA under the null hypothesis (ε0 ≤ 0.05) and alternative hypothesis (ε1 = 0.01), with a power of the not-close fit test = 0.8, model degrees of freedom = 114, and significance level = 5%. This calculation yielded a minimum required sample size of 165 [28]. The sample sizes for both men and women exceeded 165 in this study, ensuring its reliability. Statistical significance was defined as *p* < 0.05. When the test statistic difference between parameters was ≥1.96, the result was considered statistically significant. Statistical analysis was conducted by IBM SPSS Statistics for Windows Version 29.0 (IBM Corp., Armonk, NY, USA).

## 3. Results

Table 2 shows the characteristics of the participants according to sex. Obesity prevalence (BMI ≥ 25) was highest among men (32.2%) compared to women (17.1%), while women had the highest rate of being underweight (BMI < 18.5), at 10.6%, compared to men (3.9%) (*p* < 0.001).

Table 3 presents the results regarding differences in healthy diets and the related factors (each total score) according to sex and age groups. Total scores for healthy diets and maintaining an appropriate weight were significantly higher for women [*F*(1, 573) = 14.019, *p* < 0.001] [*F*(1, 573) = 49.308, *p* < 0.001]; for each age group, those scores were significantly higher in those aged 50–59 years than in those aged 40–49 years [*F*(1, 573) = 6.766, *p* = 0.010] [*F*(1, 573) = 6.048, *p* = 0.014]. The scores for the frequency of eating rice and eating out were significantly higher for men [*F*(1, 573) = 5.466, *p* = 0.020] [*F*(1, 573) = 23.801, *p* < 0.001]. No significant interaction was observed between sex and age group for either of these scores.

**Figure 2:**
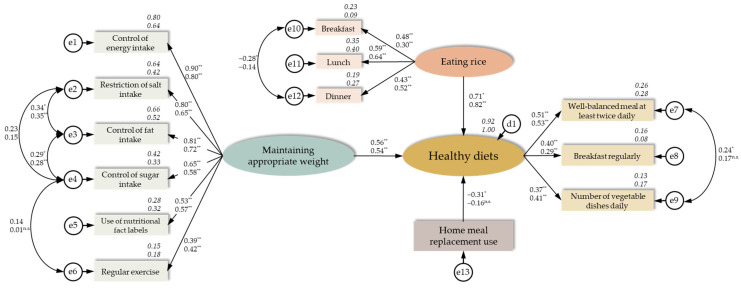
Association between healthy diets and maintaining an appropriate weight for non-communicable disease prevention, eating rice, and using home meal replacements (*n* = 577). Roman numerals in the path diagram indicate standardised estimates (next to the straight arrows) and correlation coefficients (above the bidirectional arc arrows). The numbers in italics are the *R*^2^ values (coefficients of determination). Statistical significance was set at * *p* < 0.01, and ** *p* < 0.001 (n.s., not significant). The upper number in each pair is the value for men (*n* = 255), while the lower number is that for women (*n* = 322). The results of simultaneous multi-population analysis by sex suggested that the hypothetical model had acceptable goodness-of-fit [χ^2^ = 163.657, *df* = 114 (*p* = 0.002), GFI = 0.958, AGFI = 0.933, CFI = 0.971, RMSEA = 0.028, AIC = 299.657].

We analysed an initial hypothetical model (Figure 1) using factors potentially associated with healthy diets. Consequently, we deleted eating out from the initial model, which was not significantly associated with healthy diets for both sexes, after confirming that there is no correlation between maintaining an appropriate weight and eating rice. In Figure 2, the final hypothesis model shows the association between healthy diets with maintaining an appropriate weight to prevent NCDs, eating rice, and using home meal replacements. Simultaneous multi-population analysis by sex showed acceptable goodness-of-fit indices (GFI = 0.958, AGFI = 0.933, CFI = 0.971, RMSEA = 0.028, AIC = 299.657). Maintaining appropriate weight (men: standardised estimate 0.56, *p* < 0.001; women: 0.54, *p* < 0.001) and eating rice (0.71, *p* = 0.002; 0.82, *p* < 0.001) were associated with better healthy diet scores in both men and women. However, for men, using home meal replacements was associated with lower healthy diet scores (−0.31, *p* = 0.003).

## 4. Discussion

This study investigated the structural associations, including dietary behaviours, such as eating rice and eating out/home meal replacement use, with the relationship between maintaining appropriate weight to prevent NCDs and healthy diets in middle-aged Japanese—a cohort with an increased prevalence of obesity and NCDs.

The present study showed that maintaining an appropriate weight to prevent NCDs was associated with healthy diets composed of at least two balanced meals daily, eating breakfast regularly, and eating at least five vegetable-based dishes daily in both men and women. In other words, we investigated their association with the healthy eating behaviours recommended by the WHO [3]. Among balanced meal combinations, ‘side dishes’, mainly vegetables, are the least commonly eaten [20]; however, the more the participants knew about the benefits of preventing obesity, the more vegetables they consumed [5]. Meanwhile, many of those who skipped breakfast had poor control of their weight [29,30]. These factors may have contributed to the healthy diet patterns seen in this study. Furthermore, in a population at increased risk of obesity and hypertension, actively eating potassium-rich vegetables and fruits, along with salt restriction, may prevent obesity [4]. Therefore, the association between limiting salt intake and healthy eating, one component of maintaining an appropriate weight to prevent NCDs, is probably mediated by eating vegetables.

In Japan, the 2000 Japanese food-based dietary guidelines [31] recommend a well-balanced diet consisting of a staple, main dish, and side dishes. Our findings on the positive effect of rice consumption frequency and healthy diets agreed with those of previous studies showing links between this traditional meal structure [18] and consuming breakfast [19].

Japanese cuisine traces its roots to ‘honzen ryori’ (honzen cuisine), which was used to entertain guests of samurai families in the Muromachi period (CE 1336–1573), is based on ‘ichijyu sansai’ (one soup and three dishes: vinegared, simmered, and grilled), in addition to rice and savoury dishes [32,33,34]. Throughout its extensive history, Japanese food culture has permeated the dietary habits of the Japanese people, where rice is the staple eaten alongside the ‘okazu’ (main course and side dishes). Therefore, eating rice may naturally facilitate the consumption of complete meals with main and side dishes.

Outside of Asia, a 10-year study from the United States reported that a high proportion of adults who consumed rice had better dietary quality than those who did not [17]. These findings suggest that rice may be widely considered a potential aid for sustainable healthy diets in the future, even in countries where rice is not the staple food.

The food industry has a critical role to play in promoting healthy diets, particularly by ensuring that healthy food options are available at affordable prices. Here, we also found that using home meal replacement among men was associated with lower healthy diet scores. Foods such as vegetables, which have a low caloric density, occupy a greater stomach volume than foods with high caloric density because they contain more fibre and water, resulting in a lower overall caloric intake and quick satiety [35]. Consumption of more fruits and vegetables in conjunction with dietary salt reduction may help reduce body weight and BMI [4]. The WHO recommends public food procurement and service policy interventions that limit salt or sodium-rich foods as a ‘best buy’ for reducing sodium intake and preventing or managing NCDs [36]. For example, one effective and feasible policy could involve exempting reduced-sodium and vegetable-rich dishes from sales tax, with the government reimbursing the exempted sales tax to the food service industry.

Despite its contributions, our study has some limitations. First, the participants were residents of one region in Japan—Hyogo Prefecture—and represented a relatively health-conscious population who cooperated in the 2016 Hyogo Diet Survey. Second, since this study utilises self-reported data, reporting bias may be present. Third, this study does not account for key sociodemographic variables such as household income and educational attainment. The absence of these variables introduces a risk of confounding, which must be acknowledged as a significant limitation. Fourth, the frequency of rice consumption was measured without distinguishing between white and brown rice. Since the WHO recommends increasing whole grain intake as part of a healthy diet [3], this distinction will be necessary in future research. Finally, because this was a cross-sectional study, we could not elucidate the causal relationship between maintaining appropriate weight and improved healthy diets, including the potential role of eating rice. Longitudinal studies should be conducted to clarify whether maintaining an appropriate weight, along with associated behaviours such as rice consumption, leads to improvements in diet quality. In the future, we intend to examine the effects of maintaining appropriate weight and eating rice, considering the form in which it is eaten, such as white or brown rice on sustainable healthy diets through follow-up studies addressing a larger sample of Japanese individuals.

## 5. Conclusions

Our study revealed that maintaining appropriate weight for NCD prevention may contribute to healthier diet patterns among middle-aged Japanese individual in Hyogo Prefecture, with rice consumption playing a potential role. For men, using home meal replacements was also associated with lower healthy diet scores. In the future, policies should encourage the food service industry to promote healthy eating environments by offering low-salt meals with ample vegetables and fruits in addition to other efforts to support weight management to prevent NCDs. Furthermore, the causal relationship between maintaining an appropriate weight and healthy diets, including the potential role of eating rice, needs to be further investigated using nationwide longitudinal studies and research involving non-Japanese populations.

## Figures and Tables

**Table 1:** Items related to dietary and lifestyle behaviour and their interval scales used in the analysis.

Items	Interval Scale	Score (Points)
Healthy diets		
Having a well-balanced meal at least twice daily		
	6 or 7 days/week	4
	4 or 5 days/week	3
	2 or 3 days/week	2
	1 day/week or fewer	1
Eating breakfast regularly		
	6 or 7 days/week	4
	4 or 5 days/week	3
	2 or 3 days/week	2
	1 day/week or fewer	1
Number of vegetable dishes daily		
	5 dishes or more	5
	4 dishes	4
	3 dishes	3
	2 dishes	2
	1 dish or fewer	1
Maintaining appropriate weight		
Control of energy intake		
	Daily	4
	Sometimes	3
	Not very often	2
	Never	1
Restriction of salt intake		
	Daily	4
	Sometimes	3
	Not very often	2
	Never	1
Control of fat intake		
	Daily	4
	Sometimes	3
	Not very often	2
	Never	1
Control of sugar intake		
	Daily	4
	Sometimes	3
	Not very often	2
	Never	1
Use of nutritional fact labels		
	Always	4
	Sometimes	3
	Not very often	2
	Rarely	1
Regular exercise		
	Always	4
	Sometimes	3
	Used to, but not currently	2
	Never	1
Other items that potentially relate to healthy diets		
Frequency of eating rice		
Breakfast	7 days/week	5
	5 or 6 days/week	4
	3 or 4 days/week	3
	1 or 2 days/week	2
	0 days/week	1
Lunch	7 days/week	5
	5 or 6 days/week	4
	3 or 4 days/week	3
	1 or 2 days/week	2
	0 days/week	1
Dinner	7 days/week	5
	5 or 6 days/week	4
	3 or 4 days/week	3
	1 or 2 days/week	2
	0 days/week	1
Eating out frequency		
	At least twice daily	7
	Once daily	6
	4 to 6 days/week	5
	2 or 3 days/week	4
	1 day/week	3
	1–3 days/month	2
	Not at all	1
Frequency of home meal replacement (ready-to-eat food) use		
	At least twice daily	7
	Once daily	6
	4 to 6 days/week	5
	2 or 3 days/week	4
	1 day/week	3
	1–3 days/month	2
	Not at all	1

**Table 2:** Characteristics of participants (*n* = 577).

Characteristics	Total		Men		Women		*p*
	*n* = 577		*n* = 255		*n* = 322		
	*n*	%	*n*	%	*n*	%	
Age							
40–49 years	305	52.9	132	51.8	173	53.7	0.64 ^†^
50–59 years	272	47.1	123	48.2	149	46.3	
Living arrangement							
Living alone	25	4.3	13	5.1	12	3.7	0.83 ^‡^
Married couple	95	16.5	42	16.5	53	16.5	
Parent(s) and children	352	61.0	158	62.0	194	60.2	
3- or 4-generation household	99	17.2	40	15.7	59	18.3	
Other	6	1.0	2	0.8	4	1.2	
Body mass index (BMI, kg/m^2^)							
<18.5	44	7.6	10	3.9	34	10.6	<0.001 ^†^
≥18.5 and <25	396	68.6	163	63.9	233	72.4	
≥25	137	23.7	82	32.2	55	17.1	

^†^ Chi-squared test; ^‡^ Fisher’s exact test.

**Table 3:** Differences in healthy diets and related factors (each total score) according to sex and age groups (*n* = 577).

Healthy Diets and Related Factors	Men, *n* = 255		Women, *n* = 322		Interaction (Sex × Age)	Sex		Age Group	
	40–49 Years	50–59 Years	40–49 Years	50–59 Years					
	*n* = 132	*n* = 123	*n* = 173	*n* = 149					
	Mean	Mean	Mean	Mean					
	SD	SD	SD	SD					
Healthy diets					*F* = 0.220	*F* = 14.019	Men < Women	*F* = 6.766	40–49 < 50–59
Total score (3–13 points)	8.5	9.0	9.2	9.6	*df* = 1, 573	*df* = 1, 573		*df* = 1, 573	
	2.2	2.1	1.9	2.0	*p* = 0.64	*p* < 0.001		*p* = 0.010	
Maintaining appropriate weight					*F* = 1.012	*F* = 49.308	Men < Women	*F* = 6.048	40–49 < 50–59
Total score (6–24 points)	13.2	13.7	15.0	16.1	*df* = 1, 573	*df* = 1, 573		*df* = 1, 573	
	4.1	3.8	3.3	3.2	*p* = 0.32	*p* < 0.001		*p* = 0.014	
Frequency of eating rice					*F* = 0.229	*F* = 5.466	Men > Women	*F* = 1.277	
Total score (3–15 points)	10.1	10.2	9.5	9.8	*df* = 1, 573	*df* = 1, 573		*df* = 1, 573	
	2.6	2.5	2.3	2.5	*p* = 0.63	*p* = 0.020		*p* = 0.26	
Eating out frequency					*F* = 1.233	*F* = 23.801	Men > Women	*F* = 0.146	
Score (1–7 points)	2.7	2.5	2.1	2.2	*df* = 1, 573	*df* = 1, 573		*df* = 1, 573	
	1.2	1.3	0.7	0.9	*p* = 0.28	*p* < 0.001		*p* = 0.70	
Frequency of home meal replacement use					*F* = 0.026	*F* = 0.364		*F* = 0.215	
Score (1–7 points)	2.7	2.7	2.6	2.6	*df* = 1, 573	*df* = 1, 573		*df* = 1, 573	
	1.3	1.4	1.2	1.2	*p* = 0.87	*p* = 0.55		*p* = 0.64	

Two-way ANOVA.

## Data Availability

The data presented in this study are available on request from the corresponding author due to confidentiality reasons.

## Abbreviations

The following abbreviations are used in this manuscript:

NCD	non-communicable disease
BMI	body mass index
WHO	World Health Organization
GFI	goodness-of-fit index
AGFI	adjusted goodness-of-fit index
CFI	comparative fit index
RMSEA	root mean square error of approximation
AIC	Akaike’s information criterion
SD	standard deviation
